# Masking release by combined spatial and masker-fluctuation effects in the open sound field

**DOI:** 10.1121/1.5014053

**Published:** 2017-12-04

**Authors:** John C. Middlebrooks

**Affiliations:** Department of Otolaryngology, University of California at Irvine, Irvine, California 92697-5310, USA

## Abstract

In a complex auditory scene, signals of interest can be distinguished from masking sounds by differences in source location [spatial release from masking (SRM)] and by differences between masker-alone and masker-plus-signal envelopes. This study investigated interactions between those factors in release of masking of 700-Hz tones in an open sound field. Signal and masker sources were colocated in front of the listener, or the signal source was shifted 90° to the side. In Experiment 1, the masker contained a 25-Hz-wide on-signal band plus flanking bands having envelopes that were either mutually uncorrelated or were comodulated. Comodulation masking release (CMR) was largely independent of signal location at a higher masker sound level, but at a lower level CMR was reduced for the lateral signal location. In Experiment 2, a brief signal was positioned at the envelope maximum (*peak*) or minimum (*dip*) of a 50-Hz-wide on-signal masker. Masking was released in dip more than in peak conditions only for the 90° signal. Overall, open-field SRM was greater in magnitude than binaural masking release reported in comparable closed-field studies, and envelope-related release was somewhat weaker. Mutual enhancement of masking release by spatial and envelope-related effects tended to increase with increasing masker level.

## I. INTRODUCTION

In a typical auditory scene, listeners can detect and recognize sounds of interest (signals) in the presence of other competing sounds (maskers). This task is aided by relative fluctuations in the envelopes of signals and maskers (often referred to as “monaural” masking release) and by characteristics of interaural phase differences (IPDs), formalized in closed-field (i.e., headphone) listening conditions as “binaural masking level differences” (BMLD). Monaural and binaural factors that provide release from masking have received considerable study as independent phenomena (e.g., Hirsh, 1948; Jeffress, 1948; Durlach and Colburn, 1978; Hall *et al*., 1984). In real-world listening situations, however, monaural and binaural factors almost certainly interact. The present study explored possible interactions between such monaural and binaural factors using stimuli presented from loudspeakers positioned in front or 90° to the side of the listener.

The present work was inspired by two types of experiment using closed-field conditions that examined interactions between envelope fluctuations and BMLD. Both required listeners to detect 500- or 700-Hz tonal stimuli in the presence of narrow masker bands that were centered on the signal frequency. In those experiments, maskers were always in phase at the two ears (designated *N*_0_), and the signal was in phase (*S*_0_) or antiphase (*S_π_*) at the two ears; some studies have used signal phase relations additional to 0 and *π* (e.g., Epp and Verhey, 2009a). Improvement in thresholds in the *N*_0_*S_π_* condition compared to *N*_0_*S*_0_ thresholds was interpreted as BMLD. The two types of experiment differed with regard to the presence of energy in the masker spectra in flanking frequency bands and in the emphasis in the experimental design given to details of time-domain envelopes.

One of those types of experiments measured BMLD within the context of classical comodulation masking release (CMR) (Hall *et al*., 1984). That is, detection of a tone was measured in conditions in which the envelopes of flanking noise bands were correlated or uncorrelated with the on-signal band and with each other; those were the Comodulated (CM) or Uncorrelated (UN) conditions, respectively. A lower threshold in the CM condition relative to the UN condition was taken as CMR. This is the UN–CM form of CMR, which was used in the present study and, for instance, in those by Epp and Verhey (2009a,b); this could be considered “across-band” CMR (Carlyon *et al*., 1989) because the ∼1/2 octave separation between on-channel and flanking bands minimized within-channel effects. This differs from other studies that compared the CM condition to a “reference” condition in which there are no flanking bands (Moore *et al*., 1990). Some of the studies of combined (across-channel) CMR and BMLD showed *additivity* of CMR and BMLD (Epp and Verhey, 2009a,b; Epp *et al*., 2013), meaning that release from masking was equal to the sum of CMR and BMLD and that the magnitudes of CMR and of BMLD were essentially independent of each other. Other such studies showed *sub-additivity*, meaning that the magnitude of CMR was reduced by out-of-phase binaural conditions and/or that BMLD was suppressed by comodulation (Hall *et al*., 1988, 2011).

The second of those two types of experiment measured BMLD with reference to time epochs corresponding to the maxima (*peaks*) or minima (*dips*) in the masker envelope. These experiments either used an analysis referred to as “conditional on single stimulus” to evaluate perceptual weights given to stimulus epochs having relatively high or low signal-to-masker ratios (Grose and Hall, 1998), or they presented a brief stimulus specifically at the time of the peak or dip of the masker envelope (Buss *et al*., 2003, 2007). Those studies both demonstrated *supra-additivity* of envelope fluctuation and binaural effects in that BMLD was enhanced when the signal was presented during a masker dip and the difference in detection thresholds between dip and peak conditions was enhanced in the *N*_0_*S_π_* condition. Similarly, BMLD has been shown to be greater for Gaussian maskers than for maskers having minimal envelope fluctuations (Eddins and Barber, 1998; Hall *et al*., 1998).

The goal of the present work was to translate the study of combined binaural and envelope-related masking release from BMLD under headphones to *spatial* release from masking (SRM) in open sound field conditions. Specifically, the masker source was fixed in location in the horizontal plane straight in front of the listener (0° azimuth), and the tonal signal was presented either at 0° azimuth (*N*_0_*S*_0_) or 90° to the listener's right side (*N*_0_*S*_90_). The signal frequency in all of the present experiments was 700 Hz. At that frequency, the maximum interaural delay imposed by the human head and ears is approximately half of the period of the tone (Kuhn, 1977), meaning that the IPD produced by a source at 90° azimuth is roughly *π* radians. In experiment 1A, stimuli were patterned after those of Epp and Verhey (2009a,b). The signal was a 700-Hz tone, and the masker consisted of five 24-Hz-wide components centered at 300, 400, 700, 1000, and 1100 Hz. In the UN condition, envelopes of the five components were mutually uncorrelated, whereas in the CM condition, envelopes of the five components were derived from a single noise sample; experiment 1A was supplemented by experiment 1B, under headphones. In experiment 2, stimuli were patterned after those of Buss *et al*. (2003). The masker was a single 50-Hz-wide noise band centered in frequency on the 700-Hz signal. The brief signal was presented at a time corresponding to a dip or peak of the masker envelope. The present study differed from that of Buss *et al*. (2003) in that those authors used a 500-Hz stimulus, whereas we used a 700-Hz stimulus to take advantage of the aforementioned relationship between the wavelength at 700 Hz and the dimensions of the human head.

The present results demonstrate robust release of masking both by envelope fluctuations and by spatial separation of signal and masker sources. In both open-field experiments, the SRM was somewhat greater in magnitude than the BMLD reported in the corresponding closed-field experiments (Buss *et al*., 2003; Epp and Verhey, 2009a,b). Experiment 1 revealed a dependence on masker level. Namely, envelope and spatial effects were approximately additive at a higher masker level, as reported for closed-field conditions by Epp and Verhey (2009a), whereas those effects were markedly sub-additive at a lower masker level. Experiment 2 confirmed the supra-additivity of envelope and SRM effects predicted by the results from Buss *et al*. (2003). Indeed, the supra-additivity appeared at a lower masker level and was somewhat greater in magnitude at both masker levels compared to the results obtained under headphones (Buss *et al*., 2003). The results suggest important interactions among spatial and envelope factors that could enhance hearing in real-world complex auditory scenes.

## II. GENERAL METHODS

All procedures were in accord with a protocol approved by the Institutional Review Board at the University of California at Irvine. There were two main experiments, 1A and 2, that used stimuli presented from loudspeakers in an open sound field. Experiment 1A was followed by a supplemental experiment, 1B, that used stimuli presented through headphones.

### A. Listeners

A total of nine paid listeners were recruited from the student body of the University of California at Irvine. Data from one of those listeners were eliminated from the analysis because of that listener's high variation across multiple measures of thresholds; for instance, a measure of the range of variation (the “inner range” described below) for that listener averaged 5.2 dB compared to averages ranging from 2.0 to 3.0 dB for each of the other eight listeners. Of the remaining eight listeners, two (L84 and L85) were members of the laboratory. None of the eight listeners had more than 1 h previous experience as a psychophysical listener. Ages ranged from 18 to 39 years (median 19.5), and five were female. The listeners were screened for audiometric thresholds of 15 dB hearing level or better at one-octave intervals from 0.25 to 8 kHz. All had thresholds that differed at the two ears by no more than 10 dB except for listener L80, who had a 15-dB difference at 8 kHz, and L84, who had a 15-dB difference at 4 kHz. All of the eight listeners passed the screening and completed all components of the study.

### B. Experimental apparatus and stimulus generation

Experiments were conducted inside a double-walled sound attenuating booth (Industrial Acoustics, Inc., North Aurora, IL) that was lined with 60-mm-thick SONEX Valueline absorbent foam (West General Acoustics, San Jose, CA). That foam provides substantial sound absorption in the frequencies of interest for this study, but the sound booth cannot strictly be claimed to be anechoic. For that reason, the stimulus conditions are referred to as “open sound field” rather than “free field.” The working space inside the foam was 2.6 × 2.6 × 2.7 m. The listener was seated with his or her head centered in the booth and interaural axis ∼1.4 m above the floor. The listener was instructed to maintain his or her head orientation toward the loudspeaker located in the front of the booth, i.e., 0° azimuth. A hand-held touch tablet (iPad, Apple, Cupertino, CA), interfaced to the personal computer with Duet software (Duet, Inc., Miami, FL) was used to indicate stimulus intervals, to record subject responses, and to provide trial-by-trial feedback. Open-field stimuli were presented via two bookshelf loudspeakers, Polk Audio Model T15, positioned with their faces 0.91 m from the center of the sound booth. One was positioned directly in front of the listener at ear level. The other was positioned at ear level 90° to the listener's right side. Closed-field stimuli were presented via Sennheiser HD-265 circumaural headphones.

Sounds were synthesized with 24-bit precision at a rate of 48 428 samples s^–1^ using System III equipment from Tucker-Davis Technologies (TDT, Alachua, FL). The TDT equipment was interfaced to a personal computer that ran custom matlab scripts (The Mathworks, Natick, MA). Responses of the loudspeakers were calibrated daily, and stimuli were adjusted to equalize outputs across frequencies and between the two loudspeakers. Calibration utilized pure-tone probes with recordings from a 1/2-in. microphone (ACO Pacific) positioned at the center of the sound booth in the absence of the listener. The headphones were calibrated similarly using a flat-plate coupler to equalize outputs across frequencies and between headphones.

Signals consisted of 700-Hz pure tones that were shaped with *brief* or *long* time windows. In all cases, a Gaussian impulse was generated having a standard deviation (s.d.) of 7.5 ms and truncated to ±4 s.d. (i.e., ±30 ms). The rise time of the Gaussian from 10% to 90% was 12.7 ms, and the duration between the 10% points of onset and offset was 32.2 ms. The brief time window consisted of that Gaussian impulse itself, whereas the long time window consisted of the Gaussian impulse convolved with a rectangular window, 250 ms in duration. The long signals were used for Experiments 1A and 1B, and the brief signals were used for Experiment 2.

Maskers all were 500 ms in duration, including 50-ms cosine-squared onset and offset ramps. Masker sound levels were 42 and 72 dB sound pressure level (SPL), which correspond to the lowest and highest of the three levels used by Buss *et al*. (2003). Those masker levels bracket the level of 60 dB SPL used by Epp and Verhey (2009a). Details of the masker spectra are given with the descriptions of the specific experiments.

### C. Procedure

Thresholds for detection of a tone in the various masking conditions were determined using a conventional three-interval forced choice adaptive procedure. The masker level and envelope conditions and the signal location or interaural phase difference were held constant throughout each adaptive track. On each trial, three intervals were marked by successive illumination of buttons on the response tablet. Two intervals (selected randomly) contained the masker alone, and the third contained the masker plus the signal. The listener tapped one of the response buttons to indicate the interval containing the signal. Trial-by-trial feedback was provided. The starting signal level for each adaptive track was 65 dB SPL for the 42-dB-SPL masker conditions and 85 dB SPL for the 72-dB-SPL masker conditions. After each trial, signal levels were adjusted following a two-down, one-up rule to estimate the signal level yielding 70.7% correct (Levitt, 1971). Signal levels were adjusted in 8-dB steps for the first two reversals in direction, then in 4-dB steps for the next two, then in 2-dB steps for the final six reversals. The threshold on each adaptive track was given by the mean of signal levels at the last six reversals. Listeners typically ran six or seven tracks in sessions lasting ∼30 min.

For each of the three experiments (i.e., 1A, 1B, and 2), each listener received training in the procedure and then completed, as practice, one adaptive track on each of ten masker-envelope, signal-location, and masker-level conditions. Then, each listener ran adaptive tracks for each of the experimental conditions, one condition after another, repeating that sequence for a total of three tracks on each condition. On conditions in which a listener's range of thresholds exceeded 3 dB, the listener ran two additional tracks. The *inner range* of thresholds for each condition was given by the simple range of three thresholds or by the range of five thresholds after excluding the highest and lowest values. Across all combinations of eight listeners and 30 conditions, 82% of inner ranges were ≤3.0 dB wide. The threshold for each listener on each condition was given by the median across all three or five tracks.

### D. Statistical analysis

Statistical procedures used the matlab Statistics and Machine Learning Toolbox. Analysis of variance (ANOVA) was performed using a mixed model, with the block of eight listeners treated as a random factor and the stimulus parameters as fixed factors.

## III. EXPERIMENT 1: COMODULATION AND SRM

Detection of a signal tone in on-signal masking noise is facilitated (i.e., thresholds are depressed) by the presence of identical envelope modulation among the on-signal and off-signal masking bands; conversely, thresholds typically are elevated when on- and off-signal masker envelopes are uncorrelated (Hall *et al*., 1984). Experiments conducted under headphones have demonstrated varied effects of CMR on BMLD. Several groups have shown that thresholds are higher in the CM *N*_0_*S_π_* condition than would be predicted from the sum of CMR and BMLD measured individually (Hall *et al*., 1988, 2011; Cohen and Schubert, 1991); that is, BMLD is weaker in the CM condition and/or CMR is weaker in the *N*_0_*S_π_* condition. In contrast Epp and Verhey (2009a,b) and Epp *et al.* (2013) showed rather striking *superposition* of CMR and BMLD, with essentially no significant interaction between CMR and BMLD and with masked thresholds that corresponded to the sum of CMR and BMLD, each measured alone.

Experiment 1 in the present study was patterned most closely after the study by Epp and Verhey (2009a,b). The expectation based on results of that study was that, in the open field (Experiment 1A), SRM would be unaffected by the present or absence of comodulation of flanking masker bands, and CMR would be unaffected by the location of the signal source. That is, CMR and SRM would superimpose. Our results in the open field produced an unanticipated level dependence in which the expected superposition of CMR and spatial release was evident at the higher masker level but CMR and SRM were sub-additive at the lower level.

### A. Experiment 1A: Open-field conditions

All masker stimuli were presented from the front (0°) loudspeaker. Signals were 700-Hz tones shaped by the long (250-ms) time window. In the *N*_0_*S*_0_ conditions, signals were added to the masker waveforms from the 0° loudspeaker. In the *N*_0_*S*_90_ condition, signals were presented from the 90° loudspeaker. Masker spectra consisted of five 24-Hz-wide noise bands: the on-signal band centered on the 70-Hz signal frequency plus flanking bands centered at 300, 400, 1000, and 1100 Hz. The nearest flanking component was centered ∼1/2 octave from the signal frequency. The noise bands each were created in the frequency domain by setting the real and imaginary components within each 24-Hz noise band to values drawn successively from a Gaussian distribution. This is the procedure used by Epp and Verhey (2009a) for their “Gaussian noise” maskers [data are shown in Fig. 7 of Epp and Verhey (2009a)]. For that reason, all our quantitative comparisons with the Epp and Verhey results will be with those data. In the CM condition, a single Gaussian sample was drawn for each sound presentation and was used for all of the five bands. In the UN condition, independent samples were used for each of the bands. The masker spectra were transformed to the time domain with the inverse Fourier transform and then were multiplied by the 500-ms masker window described in Sec. II B. In signal intervals, the 250-ms signal was centered in the masker window. Levels of all of the five masker bands were equal, all 42 or all 72 dB SPL; noise bands in the Epp and Verhey (2009a) study all were at 60 dB SPL.

**FIG. 1.**
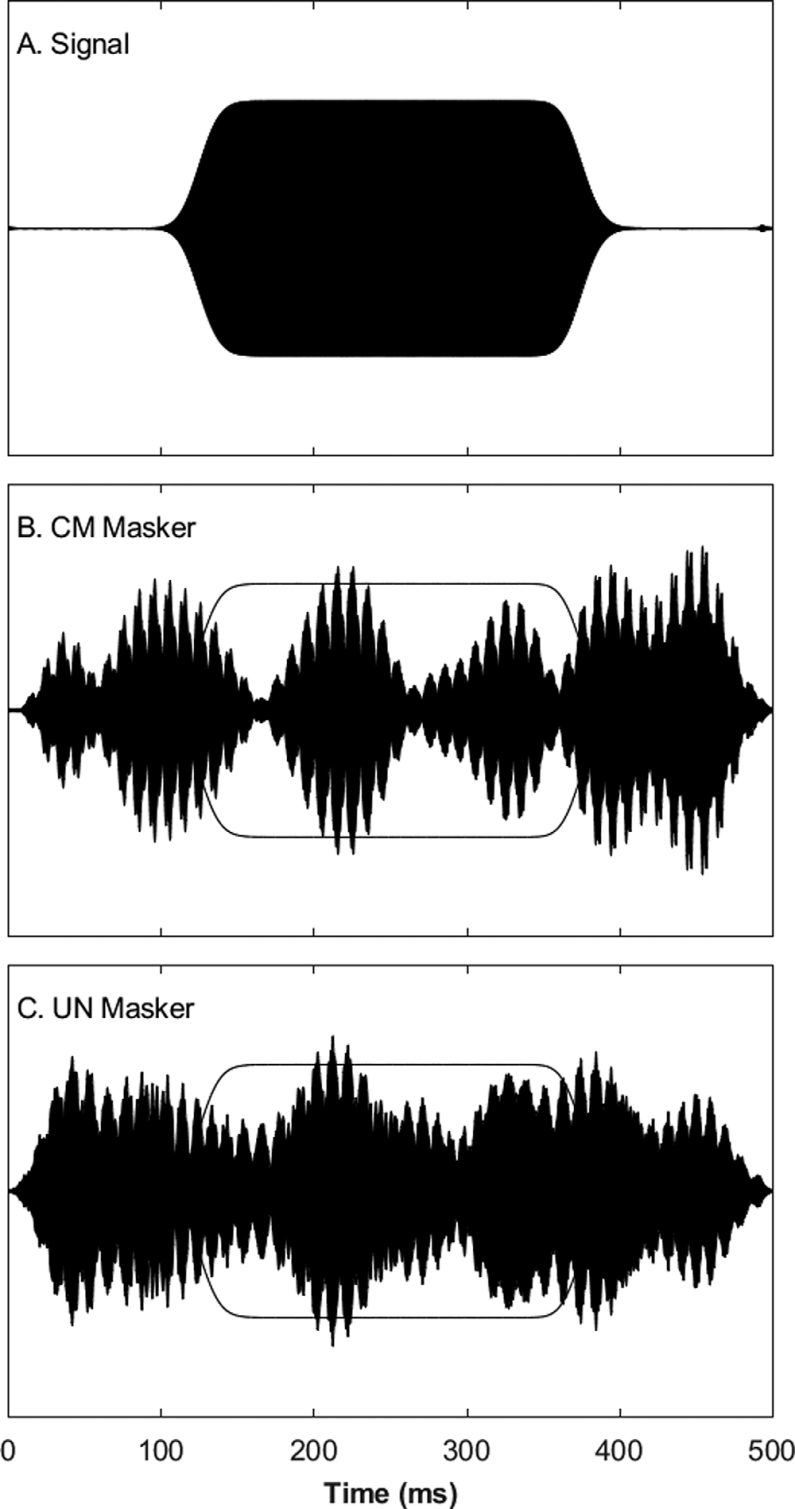
Stimulus waveforms for Experiment 1. Waveforms are shown in arbitrary pressure units. The envelope of the signal, shown in (A), is traced over the maskers, shown in (B) and (C). See text (Sec. III A) for details of stimulus synthesis.

Figure 1 shows the envelopes of the long signal [Fig. 1(A)], the CM masker [Fig. 1(B)], and the UN masker [Fig. 1(C)]; outlines of the signal envelope are duplicated in Figs. 1(B) and 1(C). These are the envelopes of waveforms recorded in the sound booth with a precision microphone in the usual position of the listener. For the purpose of illustration, one component of the UN waveform used a Gaussian sample identical to that used for the CM waveform, whereas in the actual experiments, independent samples were used for each stimulus presentation. Also, the illustrated magnitude of the signal is arbitrary, inasmuch as signal levels were adjusted adaptively from trial to trial. These waveforms show simple summation of the noise components across frequency. Note the greater depth of modulation in the CM condition, and locally higher signal-to-masker ratio, compared to the UN condition. That explicit difference in modulation depths would be informative to a listener only by broadband summation of envelopes across frequencies, for which there is no evidence in the literature. Nevertheless, the illustration provides some notion of potential benefits of utilizing envelope-related information across frequencies.

**FIG. 2.**
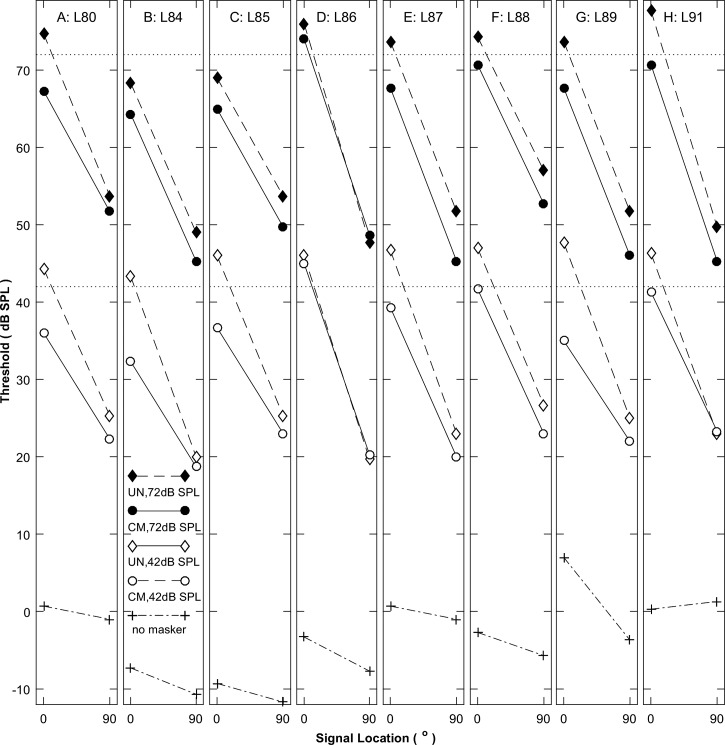
Thresholds for detection of the stimulus in various free-field masker conditions (Experiment 1) as a function of signal source location in the horizontal plane. Each panel shows median data from one listener, labeled as Lxx at the top of the panel. Filled and open symbols represent thresholds for masker levels of 72 and 42 dB SPL, respectively, and +'s represent unmasked thresholds. Diamonds and circles represent UN and CM conditions, respectively. Horizontal dotted lines show masker levels at 42 and 72 dB SPL.

Thresholds obtained in the various masker conditions are plotted in Fig. 2, one panel for each listener. The top pairs of lines (with filled symbols) represent the 72-dB-SPL masker condition, the middle pairs (with open symbols) represent the 42-dB-SPL condition, and the bottom lines (with plus signs) show the unmasked thresholds. Dashed and solid lines denote UN and CM conditions, respectively. The dotted horizontal lines indicate 72- and 42-dB-SPL masker levels. In each of the masked cases, the lines slant downward to the right, from higher threshold in the *N*_0_*S*_0_ condition to lower threshold in the *N*_0_*S*_90_ condition, indicating robust SRM. In nearly every case, the solid line in each pair was substantially lower than the corresponding dashed line, indicating the presence of CMR; the exception was listener L86, who showed negligible CMR in open field conditions. At the higher masker level (filled symbols), the solid and dashed lines are largely parallel, indicating largely constant CMR across the signal locations. This is consistent with the expectation based on the report of Epp and Verhey (2009a) that CMR magnitudes were independent of binaural condition.

At the lower masker level (Fig. 2, open symbols), the dashed lines indicating the UN condition are largely parallel to both UN and CM conditions at the higher masker level, but the solid lines indicating the CM condition are somewhat flatter, indicating reduced SRM in the CM condition and/or reduced CMR in the *N*_0_*S*_90_ condition. At the 42-dB-SPL masker level, the UN thresholds tend to approach the CM thresholds in the *N*_0_*S*_90_ condition, in some cases coming within 1 dB of the CM thresholds. That suggests that the masking release due to combined spatial and comodulation effects is compressed at low sound levels.

Previous studies have demonstrated that the magnitudes of both BMLD and CMR (studied independently) tend to decrease at low sound levels roughly corresponding to the range of thresholds that we observed in the *N*_0_*S*_90_ condition at the lower masker level. In the present study using the lower masker level, UN and CM thresholds ranged from 32.3 to 47.7 dB SPL in the *N*_0_*S*_0_ condition compared to a range of 18.7–26.7 dB SPL in the *N*_0_*S*_90_ condition (Fig. 2). Hall and Harvey (1984), for example, evaluated the BMLD for a 500-Hz tone masked by a 50-Hz-wide on-frequency noise band. The BMLDs were roughly constant in magnitude across masker levels at which thresholds were greater than about 30–40 dB SPL, like all our thresholds for the 72-dB-SPL masker level and like our *N*_0_*S*_0_ 42-dB-SPL masker condition. Hall and Harvey's (1984) BMLDs tended to decrease markedly, however, across decreasing masker levels at which signal levels were around 20 dB SPL, like our *N*_0_*S*_90_ 42-dB-SPL masker condition. Similarly, Moore and Shailer (1991) reported a ∼13-dB decrease in CMR from about 15 dB at masker levels at which signal thresholds were around 44 dB SPL (in the range of thresholds in the present *N*_0_*S*_0_ condition) down to ∼2 dB at a masker level yielding thresholds of ∼28 dB SPL, somewhat higher than the range of thresholds in the present *N*_0_*S*_90_ condition in which CMR was compressed.

A possible explanation for the present results at low masker levels is that SRM and CMR both operate through mechanisms that involve detection of a signal-induced decorrelation between stimuli at the two ears. At low stimulus levels, such mechanisms might be disrupted by the presence of a floor imposed by interaurally uncorrelated internal noise (Dolan and Robinson, 1967; Dolan, 1968; Yost, 1988). That is, in the present study, thresholds might have been high enough in the *N*_0_*S*_0_ condition to permit a benefit from comodulation comparable to that seen for the higher masker level. In contrast, in the *N*_0_*S*_90_ condition, masking release due to SRM and/or CMR might have reduced signal thresholds to a level at which internal noise precluded additional masking release by CMR or SRM.

The masked thresholds illustrated in Fig. 2 were evaluated with an ANOVA, which showed significant main effects of signal location (0 or 90°; *F*_(1,29)_ = 293, *p* < 0.001), across-band modulation correlation (CM or UN; *F*_(1,29)_ = 45, *p* < 0.001), and masker level (42 or 72 dB SPL; *F*_(1,29)_= 5156, *p* < 0.001). There was a two-factor interaction between signal location and across-band modulation correlation (*F*_(1,29)_ = 17, *p* < 0.001). The unmasked thresholds illustrated in Fig. 2 averaged 3.3 dB lower at the 90° signal location than at the 0° location (*F*_(1,7)_ = 7.5, *p* = 0.029).

**FIG. 3.**
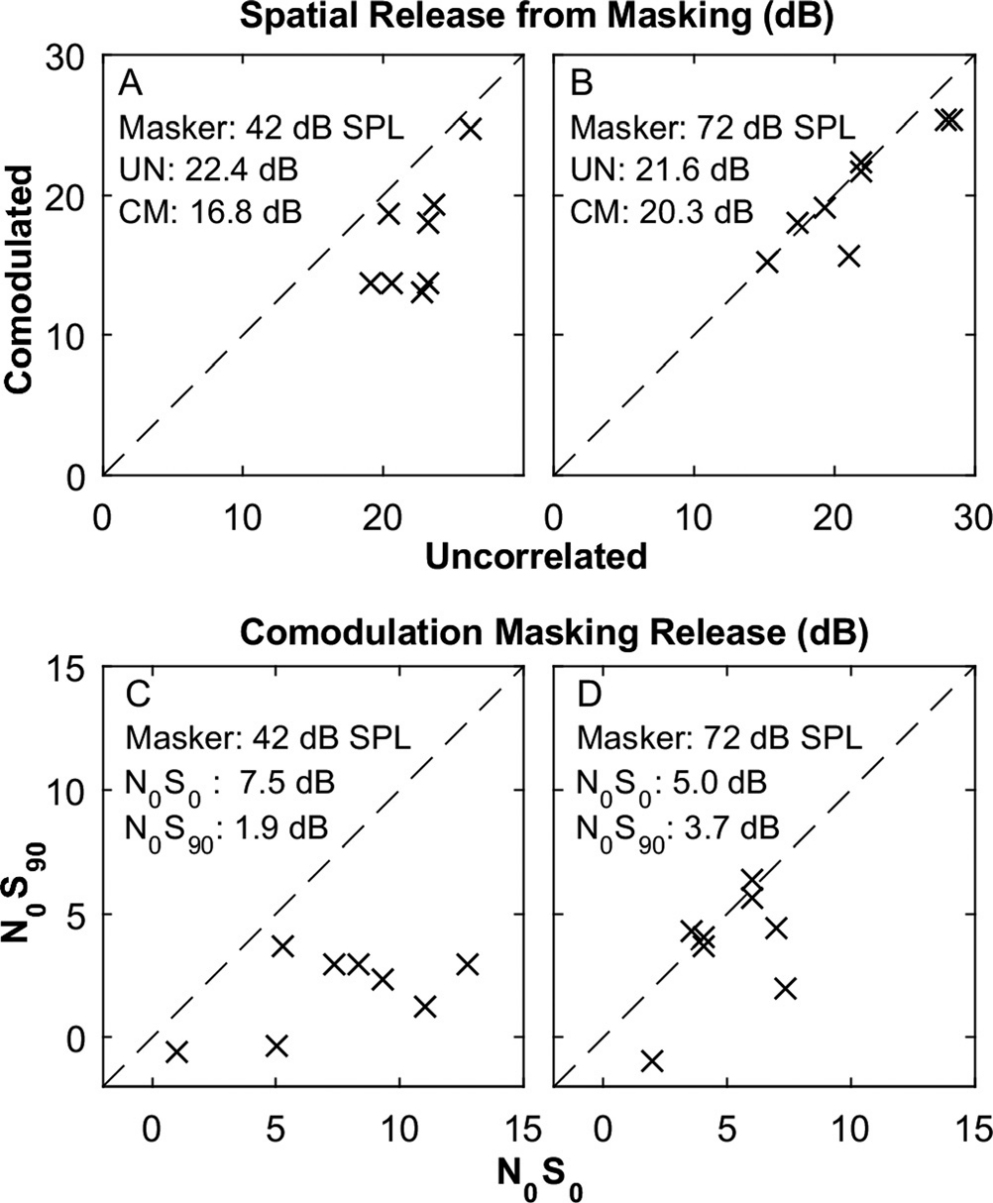
Free-field SRM (A) and (B), and CMR (C) and (D). Left and right columns of panels show results for masker levels of 42 and 72 dB SPL, respectively. In each panel, each symbol represents median data for one listener. The numerical values are means across listeners for the various conditions. (A) and (B): SRM is shown in UN (abscissa) and CM (ordinate) modulation conditions. (C) and (D): CMR in *N*_0_*S*_0_ (abscissa) and *N*_0_*S*_90_ (ordinate) conditions.

The magnitudes of SRM are shown in Figs. 3(A) and 3(B) for the CM (shown on the ordinate) and UN (abscissa) conditions; panels A and B represent data from 42- and 72-dB-SPL maskers, respectively, and symbols indicate individual listeners. Results differed between the higher and lower masker levels. At the higher level [Fig. 3(B)], most of the symbols lie near the diagonal line that represents little or no influence of comodulation on SRM (*F*_(1,7)_ = 3.0, *p* = 0.13). At the lower masker level [Fig. 3(A)], in contrast, SRM was consistently reduced in the CM compared to the UN condition (*F*_(1,7)_ = 25, *p* = 0.0015). At the lower level, the reduced SRM in the CM condition corresponds with the reduced CMR in the *N*_0_*S*_90_ condition seen in the thresholds plotted in Fig. 2. An ANOVA showed a main effect on SRM of across-band modulation correlation (*F*_(1,7)_ = 31, *p* < 0.001). There was no significant main effect of masker level on SRM (*F*_(1,7)_ = 2.1, *p* = 0.20), but there was a significant interaction between across-band modulation correlation and masker level (*F*_(1,7)_ = 8.6, *p* = 0.022).

The magnitudes of CMR are shown in Figs. 3(C) and 3(D) for the *N*_0_*S*_90_ (ordinate) compared to the *N*_0_*S*_0_ (abscissa) condition. The CMR was computed as the threshold for the UN condition minus that for the CM condition, both with flanking bands present. In the higher-masker condition [Fig. 3(D)], five of the symbols lay near the diagonal line that represents equal CMR regardless of signal location, with only three points lying appreciably below the line. At that masker level, there was no significant difference in CMR associated with signal location (*F*_(1,7)_ = 3.0, *p* = 0.13). At the lower masker level [Fig. 3(C)], however, the data consistently lay below the diagonal line, indicating significantly reduced CMR in the *N*_0_*S*_90_ condition (*F*_(1,7)_ = 25, *p* = 0.0015). The mean CMR in the *N*_0_*S*_0_ condition was somewhat higher at the lower masker level (7.5 dB) than at the higher level (5.0 dB), but a *post hoc* test of the CMR for high- versus low-level masker in the *N*_0_*S*_0_ condition failed to show a significant difference between masker levels (paired *t-*test, *t*_7_ = 2.06, *p* = 0.079).

The *combined* masking release given by comodulation and 90° separation of signal and masker is given by the threshold in the UN *N*_0_*S*_0_ condition minus that in the CM *N*_0_*S*_90_ condition. That value averaged 25.3 dB (s.d. = 4.3 dB) for the 72-dB masker level and 24.3 dB (s.d. = 1.6 dB) for the 42-dB level. That can be compared with the CMR in the *N*_0_*S*_0_ condition *plus* the SRM in the UN condition, which averaged 26.7 dB (s.d. = 5.2 dB) at the higher masker level and 29.9 dB (s.d. = 3.5 dB) at the lower level. Masking release by CMR and SRM were nearly additive at the higher masker level in that the combined masking release was only 1.3 dB less than the sum of CMR and SRM. Release by CMR and SRM was appreciably sub-additive at the lower masker level in that the combined release was 5.6 dB lower than the sum of CMR and SRM. The difference between combined and summed releases differed significantly between masker levels (*t*-test: *t*_7_ = 2.9, *p* = 0.022).

That the combined values were so similar between the higher and lower masker levels (i.e., 25.3 and 24.3 dB, respectively) may seem surprising, given the compressed CMR in the *N*_0_*S*_90_ condition at the lower level. At the lower masker level, however, the compressed CMR in the *N*_0_*S*_90_ condition is compensated somewhat by expanded CMR in the *N*_0_*S*_0_ condition.

The present open-field results at the higher masker level agree with the results of Epp and Verhey (2009a,b) in showing superposition of CMR and SRM; that is, the magnitude of CMR was largely independent of signal position and the magnitude of binaural (in this case spatial) MLD was largely independent of comodulation. Superposition was not seen, however, at the lower masker level in that CMR was reduced in the open-field *N*_0_*S*_90_ condition and, equivalently, SRM was reduced in the CM condition. Again, masking release at the lower masker level might have reached a floor determined by interaurally uncorrelated internal noise (Yost, 1988).

The present results at both masker levels agree reasonably well with those of Epp and Verhey (2009a,b) with regard to the mean magnitudes of combined masking release due to comodulation and binaural/spatial condition: ∼22 dB in the previous closed-field results, 24.3 dB at our 42-dB masker level, and 25.3 dB at our 72-dB masker level. Nevertheless, the composition of those combined releases differed between the two studies. That is, magnitudes of CMR in our study were substantially lower, averaging 1.9 to 7.5 dB depending on conditions, compared to ∼9–10 dB in the Epp and Verhey (2009a,b) results. Our SRM (averaging from 16.8 to 22.4 dB), then, was correspondingly higher than the closed-field BMLD (12–13 dB). One possible explanation for the greater masking release due to spatial signal/masker separation is that, in the open field, the proximal stimuli at the listener's ears delivered by the signals at 0° and 90° differed not only in their IPDs (0 versus ∼*π* radians) but also in their signal-to-masker ratios and their interaural level differences (ILDs). One can estimate those values from the measurements made from a KEMAR acoustic mannequin and made available on line by Gardner and Martin (1995). Those estimates show that the signal level at 700 Hz, and hence the signal-to-masker ratio, would be 2.6 dB higher at the near ear from the 90° than from the 0° loudspeaker position and that the ILD from 90° would be about 4.2 dB compared to ∼0 dB from 0°; the estimated 2.6-dB higher near-ear level at 90° accords with the unmasked thresholds averaging 3.3 dB lower at that signal location as shown in Fig. 2. It might be that those sound-level effects contributed to lower masked thresholds and greater SRM in the present *N*_0_*S*_90_ results compared to the *N*_0_*S_π_* thresholds reported by Epp and Verhey (2009a,b); it is not obvious, however, why ILD would influence UN more than CM conditions. Masking release by interaural phase and level differences was explored independently under headphones in Experiment 1B.

### B. Experiment 1B: Closed-field (headphone) conditions

Stimulus conditions in Experiment 1B were identical to those in Experiment 1A except that stimuli were presented under headphones and only the 72-dB-SPL masker level was tested. Maskers were diotic (*N*_0_; identical stimuli at the two ears) and signals were either diotic (in the *N*_0_*S*_0_ condition) or had their phase inverted between the two ears (*N*_0_*S_π_*). Conditions were added in which a 5-dB ILD in the signal was introduced by increasing the signal level at the right ear by 2.5 dB and decreasing the signal level at the left ear by 2.5 dB; that means that in those 5-dB ILD conditions, the signal level and the signal-to-masker ratio at the right ear were increased by 2.5 dB above the nominal values.

**FIG. 4.**
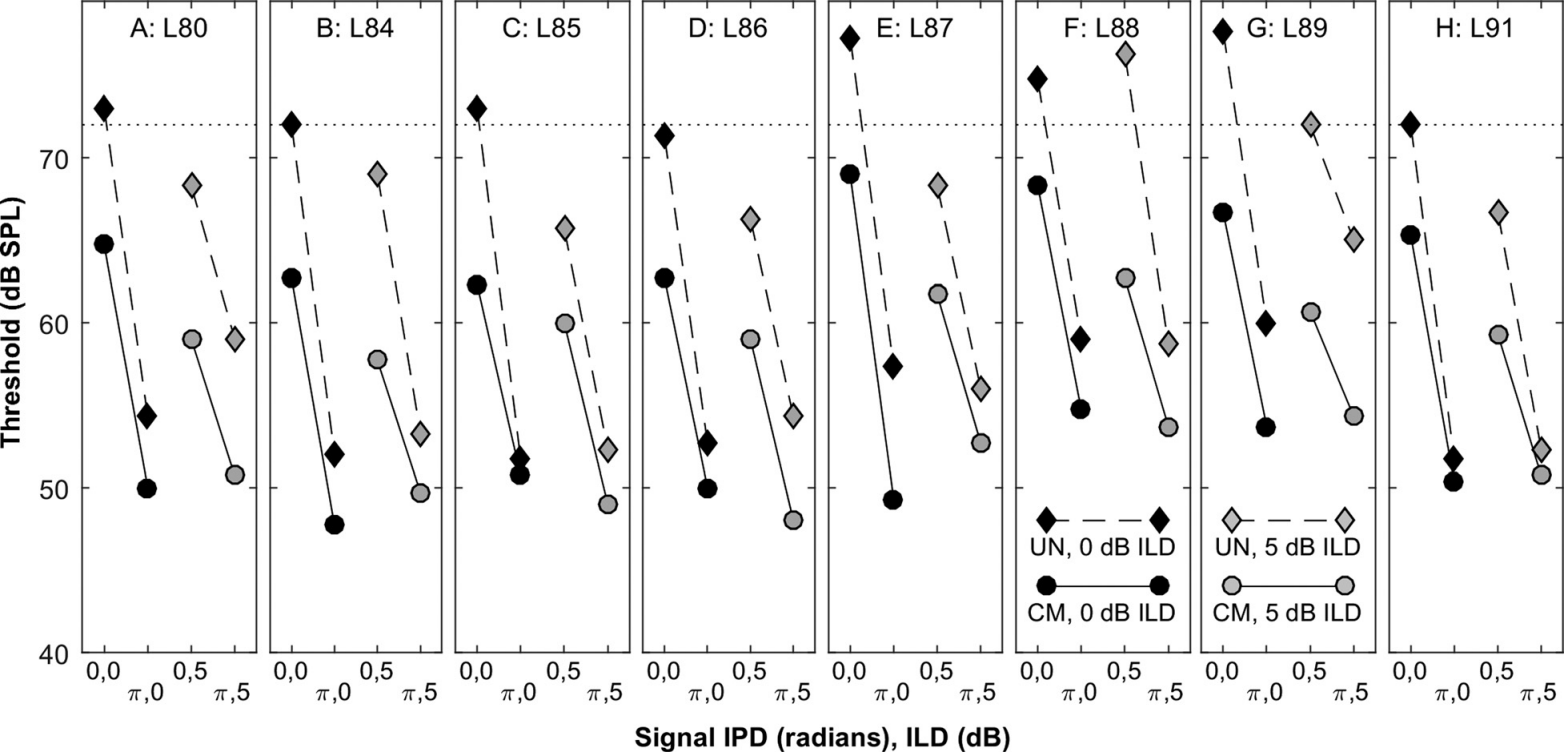
Thresholds for detection of the stimulus in various closed-field masker (symbol types) and signal (abscissa) conditions. Each panel shows data from one listener. Paired abscissa labels indicate signal IPD and ILD as 0 or *π* radian IPD, and 0 or 5 dB ILD. Black and gray fills indicate 0 and 5 dB ILD, respectively. Diamonds and circles represent UN and CM conditions, respectively. Masker levels were fixed at 72 dB SPL.

Thresholds obtained in the presence of the 72-dB-SPL masker are shown in Fig. 4. The pair of lines with black-filled symbols in the left half of each panel represents the conditions in which there was zero ILD; these were the conditions closest to that in the studies by Epp and Verhey (2009a,b) and Epp *et al*. (2013). Thresholds consistently were lower in the CM than in the UN condition, indicating the presence of CMR; that was true even for listener L86 [Fig. 4(D)] who showed negligible CMR in the open-field condition [Fig. 2(D)]. Thresholds were consistently lower in the *N*_0_*S_π_* compared to the *N*_0_*S*_0_ condition, indicating the presence of BMLD.

The pair of lines with gray-filled symbols in the right half of each panel in Fig. 4 represents the conditions in which a 5-dB ILD was introduced. As in the 0-dB-ILD condition, both CMR and BMLD were present. In the 0-radian IPD conditions, addition of 5 dB ILD almost always resulted in a substantially decreased threshold (i.e., compare *N*_0_*S*_0,0_ with *N*_0_*S*_0,5_ for both UN and CM conditions; mean difference: 5.0 dB; significant main effects of ILD, *F*_(1,7)_ = 65, *p* < 0.001 and across-band modulation correlation, *F*_(1,7)_= 290, *p* > 0.001). That was not the case in the *π*-radian condition, as addition of the 5-dB-ILD often resulted in an *elevation* of threshold. Across all listeners, however, there was no significant change in threshold associated with the introduction of 5 dB ILD in the *N*_0_*S_π_* condition; compare *N*_0_*S_π,_*_0_ with *N*_0_*S_π,_*_5_ for both UN and CM conditions. There was a significant main effect of across-band modulation correlation (*F*_(1,7)_ = 173, *p* < 0.001), but no main effect of ILD (*F*_(1,7)_ = 3.5, *p* = 0.10).

**FIG. 5.**
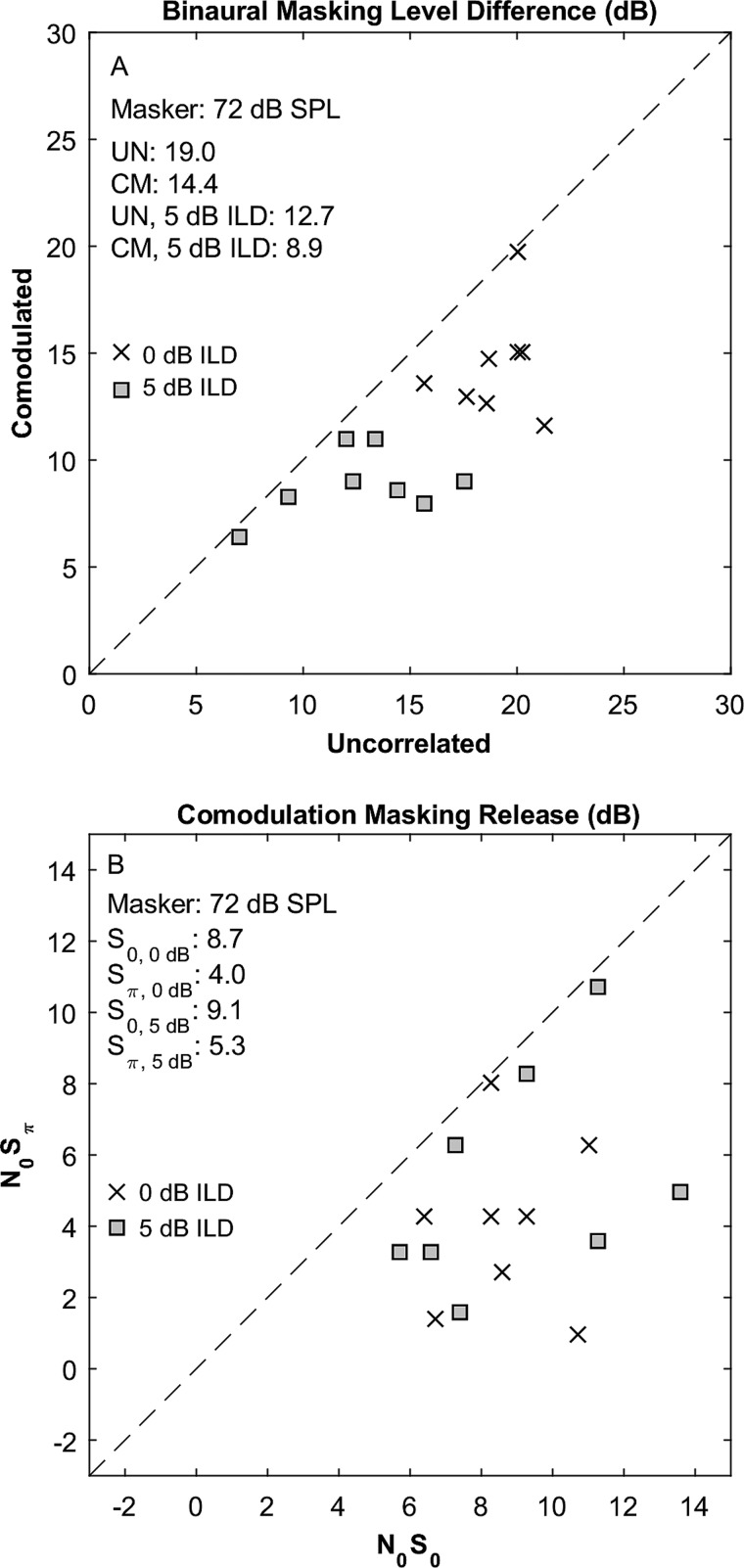
Closed-field BMLD (A) and CMR (B). Comodulation conditions are shown on the axes in (A), and binaural conditions are shown on the axes in (B). × and square symbols indicate 0- and 5-dB ILD, respectively.

The magnitudes of BMLD are shown in Fig. 5(A) for the CM (ordinate) and UN (abscissa) conditions. Symbols indicate individual listeners, with ×'s denoting the 0-dB-ILD condition, and gray-filled squares denoting 5 dB ILD. We focus first on the 0-dB ILD data. Nearly all of those closed-field BMLDs were smaller than the open-field SRMs for corresponding listeners and across-band modulation correlation conditions (paired *t*-test: *t*_15_* = *3.6, *p* = 0.0024). In Fig. 5(A), all of the data lie below the diagonal line, indicating that BMLD was decreased in the CM condition compared to the UN condition. The difference between BMLD obtained in CM versus UN conditions ranged from negligible (i.e., 0.3 dB) to substantial (9.7 dB); in the 0-ILD condition; BMLDs averaged 4.6 dB lower in the CM condition. The observed sub-additivity of comodulation and BMLD agrees with the present open-field results at the lower (42-dB-SPL) masker level but conflicts with our open-field results at the higher masker level and with the finding by Epp and Verhey (2009a,b) of additivity (i.e., superposition) of comodulation and BMLD. The difference with the Epp and Verhey results might be due to the difference in masker levels that were used, 60 dB SPL by Epp and Verhey (2009a,b) compared to 72 dB SPL in the present study, although that explanation is unsatisfying given our results in the open field showing that higher masker levels tended to move the results toward additivity rather than toward sub-additivity. Epp and Verhey (2009a) reported BMLD averaging 12–13 dB for both CM and UN conditions. The present BMLD values in the 0-dB ILD condition, 14.4 dB in CM, and 19.0 in UN, were somewhat larger than the Epp and Verhey values.

In the present study, addition of a 5-dB ILD consistently reduced the BMLD due to IPD (i.e., *N*_0_*S*_0,5dB_ minus *N*_0_*S_π_*_,5dB_ was less than *N*_0_*S*_0,0dB_ minus *N*_0_*S_π_*_,0 dB_) in both CM and UN conditions [Fig. 5(A)]. The reduced BMLD in the presence of 5 dB ILD can be accounted for by the lowering of thresholds in the 0-radian IPD conditions and the lack of consistent threshold reduction in the *π*-radian conditions. An ANOVA of all the closed-field BMLD results showed significantly reduced BMLD in the CM condition (*F*_(1,7)_ = 44, *p* < 0.001) and, notably, lower BMLD in the 5-dB-ILD condition (*F*_(1,7)_ = 40, *p* < 0.001). These data do not support the hypothesis that the enhanced MLD in the open-field compared to closed-field condition was due to ILD or to enhanced signal-to-masker ratio produced by the signal at 90° azimuth.

The magnitudes of CMR are shown in Fig. 5(B) for *N*_0_*S_π_* (ordinate) versus *N*_0_*S*_0_ (abscissa) conditions; *x*'s and squares represent conditions in which the ILD was 0 or 5 dB, respectively. All of the data lie below the diagonal line, indicating that CMR consistently was weaker in the *N*_0_*S_π_* condition. An ANOVA showed a significant main effect of IPD (*F*_(1,7)_ = 44, *p* < 0.001) and no main effect of ILD (*F*_(1,7)_ = 1.0, *p* = 0.35). The present CMR values averaged from 4.0 to 9.1 dB, depending on IPD and ILD. These CMR values were significantly higher than the present results in the open field (paired *t-*test: *t*_15_* = *2.7, *p* = 0.016) but still were somewhat lower than the values of 9–10 dB observed by Epp and Verhey (2009a).

## IV. EXPERIMENT 2: INFLUENCES OF SHORT-TERM MASKER ENVELOPE ON SRM FOR BRIEF TONES

In previous closed-field experiments using narrowband maskers centered on the signal frequency, masker envelope fluctuations appeared to enhance tone detection in *N*_0_*S_π_* conditions but not in *N*_0_*S*_0_ conditions. That is, thresholds for detection of a tone in *N*_0_*S*_0_ conditions are about equal in maskers having greater or lesser envelope fluctuations, whereas thresholds in *N*_0_*S_π_* conditions are substantially lower in the presence of deeper fluctuations (Hall *et al*., 1998). Also, listeners give greater weight to short-term minima in masker envelopes in *N*_0_*S_π_* but not *N*_0_*S*_0_ conditions (Hall *et al*., 1998). As a result, BMLD is enhanced by increases in the depth of masker envelope fluctuations.

Experiment 2 in the present study was patterned most closely after a study by Buss *et al*. (2003). In that work, a brief 500-Hz tone signal was masked by a 50-Hz-wide noise band centered on the signal frequency. The signal, 30 ms in duration, was centered in the 409-ms masker duration. The masker was shifted in time such that the signal fell at the overall minimum (referred to here as the *dip* condition) or maximum (the *peak* condition) of the masker envelope. Buss *et al*. (2003) found that in the *N*_0_*S*_0_ condition, thresholds were about equal between dip and peak conditions. In the *N*_0_*S_π_* condition, dip and peak thresholds also were about equal for most listeners at the lowest masker level (42 dB SPL), but at the highest masker level (72 dB SPL), thresholds were substantially lower for dip than peak. Equivalently, BMLD was enhanced at the dip compared to the peak, particularly at the highest masker level. The expectation in the present Experiment 2 was that SRM obtained by displacing the signal source to 90° azimuth would be enhanced in the dip compared to the peak condition.

That expectation was largely confirmed. The SRM was enhanced in the dip condition. Also consistent with the result by Buss *et al*. (2003), that effect was greater at the higher masker level, although we observed significant synergy between spatial and masker-timing effects even at the lower level.

### A. Experiment 2: Open-sound-field conditions

The signal in Experiment 2 was a 700-Hz tone multiplied by the brief (Gaussian impulse) time window. A signal frequency of 700 Hz was chosen rather than the 500 Hz signal employed by Buss *et al*. (2003) because a 700-Hz signal presented at 90° azimuth produces an IPD closer to *π* radians. Signals were presented from the 90° loudspeaker in the *N*_0_*S*_90_ condition and were added to the masker waveforms from the front (0°) loudspeaker in the *N*_0_*S*_0_ condition. The masker spectrum consisted of a single frequency band, 50 Hz wide, centered on the 700-Hz signal frequency. It was created in the frequency domain by setting the real and imaginary components within the noise band to values drawn successively from a Gaussian distribution that was independent between stimulus presentations. The masker spectrum was transformed to the time domain using the inverse Fourier transform. The envelope was extracted using the Hilbert transform, and the time point corresponding to the envelope minimum was identified. Copies of the waveform were concatenated before and after the original waveform, and then a 500-ms section was extracted centered on the time of the envelope minimum. That 500-ms section then was shaped with 50-ms cosine-squared onset and offset ramps to form the dip masker. A similar procedure was followed to form the peak masker centered on the envelope maximum. Again, masker sound levels were 42 and 72 dB SPL, corresponding to the lowest and highest of the three levels used by Buss *et al*. (2003). The signal was centered in time on the masker duration so that it fell on the envelope minimum or maximum.

**FIG. 6.**
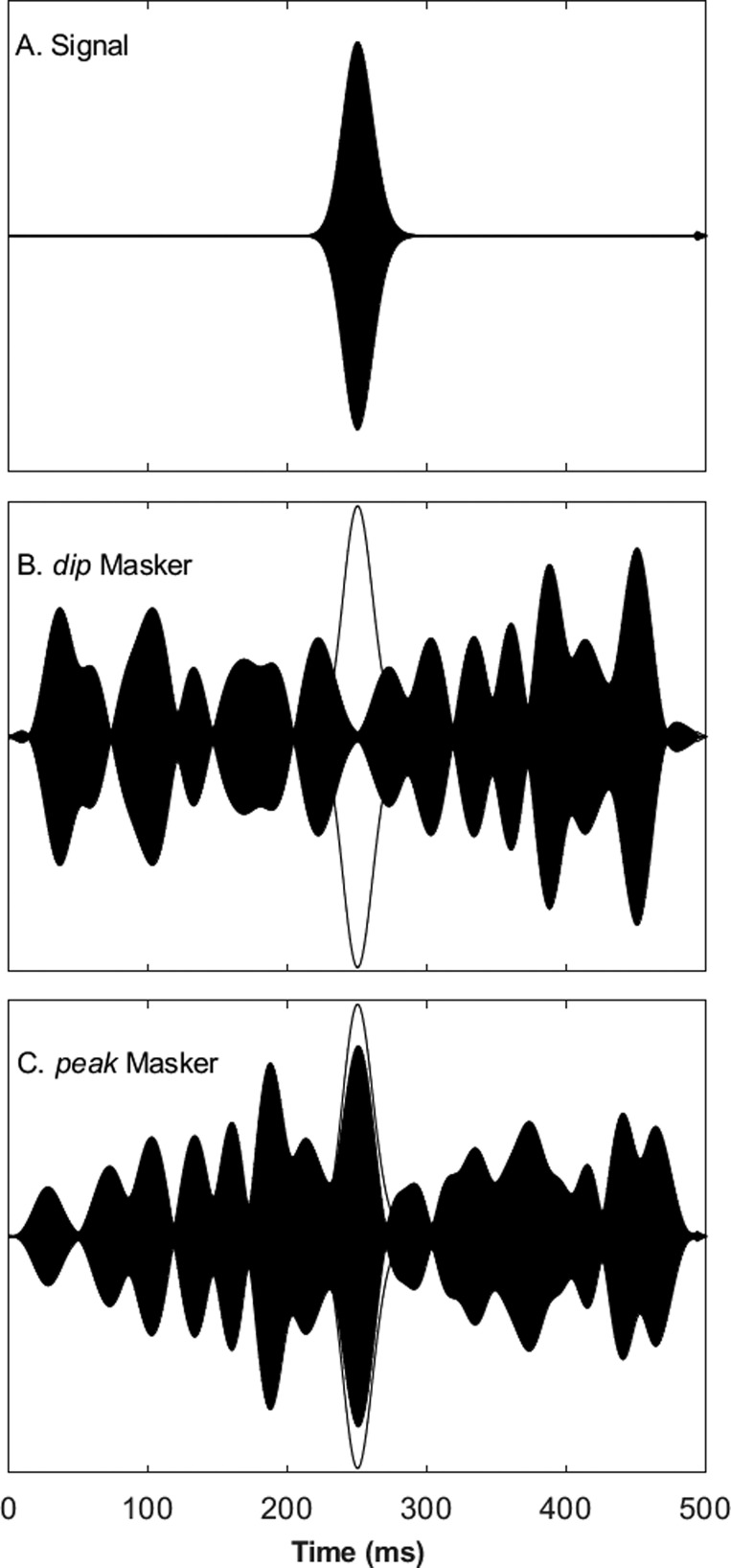
Stimulus waveforms for Experiment 2. Waveforms are shown in arbitrary pressure units. For purpose of illustration, the waveform envelopes in (B) and (C) are identical, but shifted in time; in actual experiments, each stimulus presentation utilized a new random stimulus envelope. The outline of the signal envelope in (A) is duplicated in (B) and (C). See text (Sec. III A) for details of stimulus synthesis.

Figure 6 shows the relevant waveforms recorded in the sound booth. For the purpose of illustration, the two maskers in the example were based on identical Gaussian samples, although in actual experiments, samples were independent from trial to trial. In the figure, one can see that the masker in the dip condition [Fig. 6(B)] is shifted ∼200 ms later relative to the masker in the peak condition, such that the maximum in Fig. 5(C) at 250 ms appears at ∼450 ms in Fig. 6(B).

**FIG. 7.**
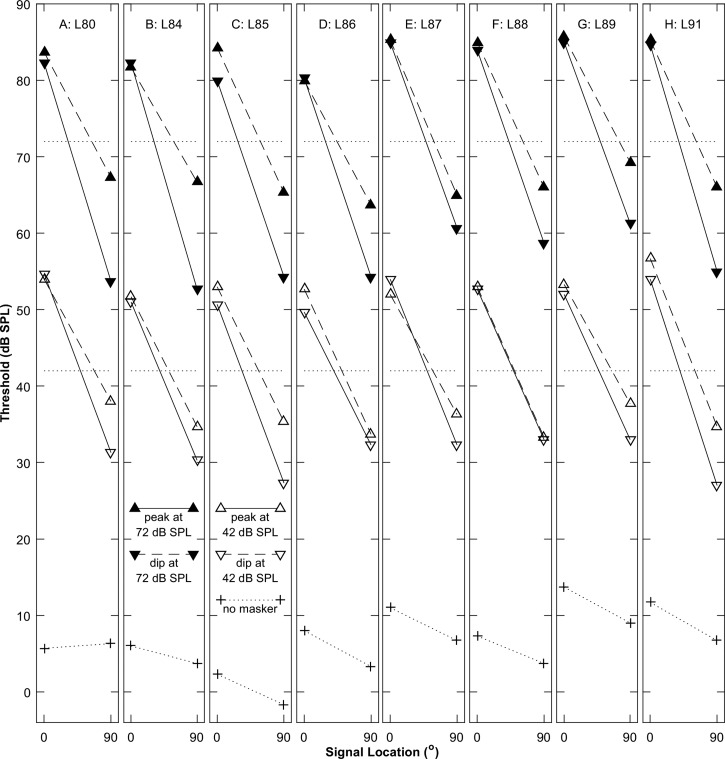
Thresholds for detection of the stimulus in various free-field masker conditions (Experiment 2) as a function of signal source location in the horizontal plane. Upward triangles and downward triangles represent conditions of signal placement at masker maximum (peak) and minimum (dip), respectively. +'s indicate the unmasked condition. All other conventions are as in Fig. 2.

Thresholds obtained in the open field with the dip and peak maskers are shown in Fig. 7, one listener per panel. The top pairs of lines (with filled symbols) represent the 72-dB-SPL masker condition, the middle pairs (open symbols) represent the 42-dB-SPL condition, and the bottom lines (plus signs) show the unmasked thresholds. At the two masker levels, upward- and downward-pointing triangles represent peak and dip conditions, respectively. In every case, the lines slope down to the right, from *N*_0_*S*_0_ to *N*_0_*S*_90_ conditions, indicating substantial SRM. At the 0° signal location, thresholds for peak and dip conditions tended to be very similar, with thresholds across both masker levels averaging only 0.9 dB higher in the peak than in the dip condition (main effect of masker timing for 0° signal: *F*_(1,22)_ = 4.55, *p* = 0.044). The dashed line representing the peak condition lies above and with flatter slope relative to the solid dip line for all listeners at the higher masker level and all but L87 and L88 at the lower level. That indicates a greater benefit of the dip condition at the 90° signal location or, equivalently, greater SRM in the dip condition. An ANOVA showed significant main effects of signal location (*F*_(1,29)_ = 1225, *p* < 0.001), masker timing (*F*_(1,29)_ = 49, *p* < 0.001), and masker level (*F*_(1,29)_ = 3384, *p* < 0.001), and significant two-factor interactions between signal location and masker timing (*F*_(1,29)_ = 60, *p* < 0.001), signal location and masker level (*F*_(1,29)_ = 9.3, *p* = 0.0050), and masker timing and masker level (*F*_(1,29)_ = 10.0, *p* = 0.0036).

**FIG. 8.**
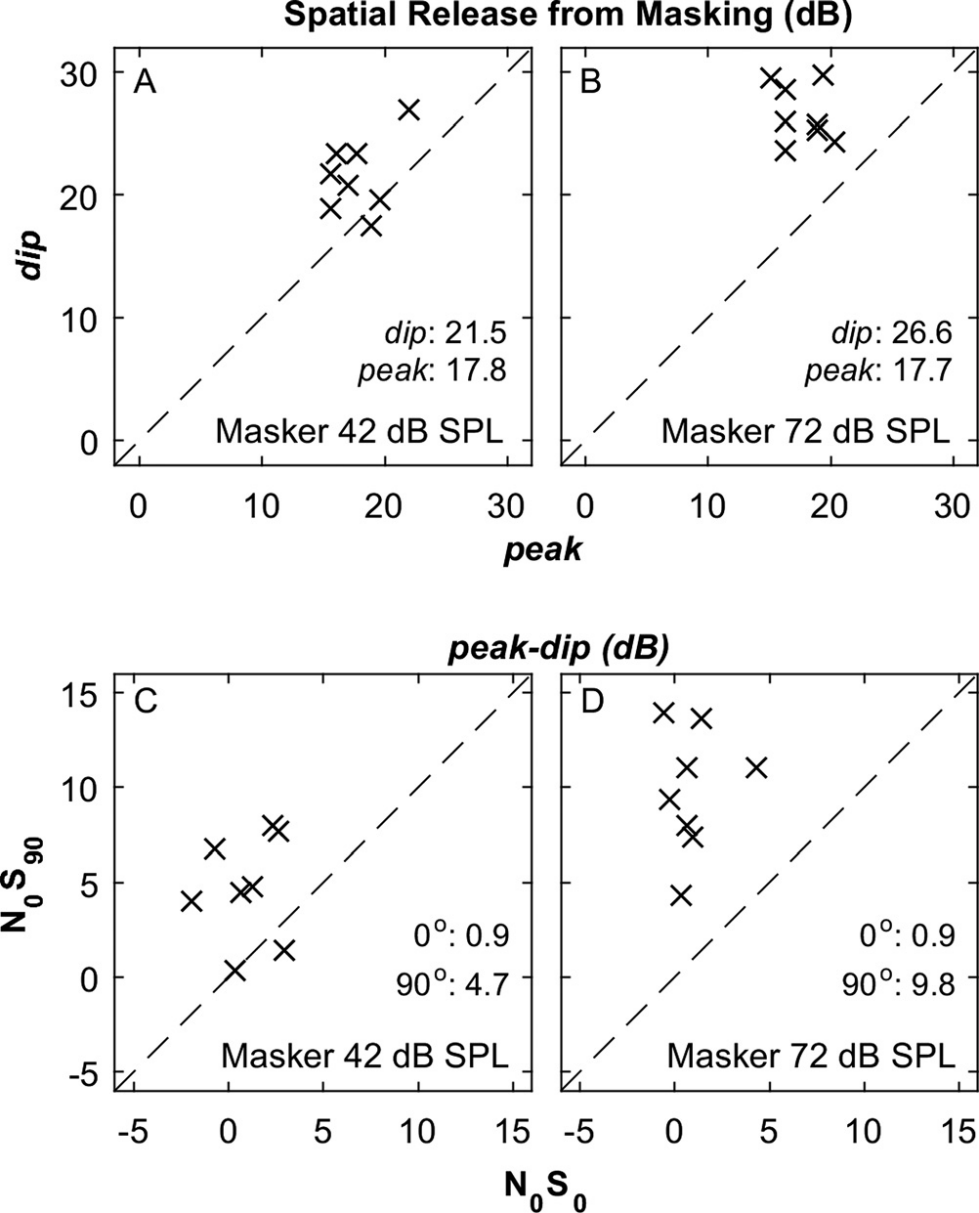
Free-field SRM (A) and (B) and peak minus dip (C) and (D). Left and right columns of panels show results for masker levels of 42 and 72 dB SPL, respectively. The numerical values are means across listeners for the various conditions. (A) and (B): SRM is shown in peak (abscissa) and dip (ordinate) modulation conditions. (C) and (D): peak*–*dip threshold differences in *N*_0_*S*_0_ (abscissa) and *N*_0_*S*_90_ (ordinate) conditions. Other conventions are as in Fig. 3.

The magnitudes of SRM are shown in Figs. 8(A) and 8(B) for the dip and peak conditions; the left and right panels represent data from 42- and 72-dB-SPL maskers, and symbols indicate individual listeners. Most of the symbols lie above the diagonal lines, indicating greater SRM in the dip condition. The SRM was nearly equal between masker levels in the peak condition, 17.7 or 17.8 dB, whereas in the dip condition, SRM was appreciably greater at the higher masker level (26.6 compared to 21.5 dB). An ANOVA showed significant main effects of masker timing (*F*_(1,7) = _56, *p < *0.001) and of masker level *F*_(1,7)_ = 37, *p* < 0.001), and a significant two-factor interaction between masker timing and masker level (*F*_(1,7)_ = 10.6, *p* = 0.014).

Figures 8(C) and 8(D) plot the difference between peak and dip thresholds in *N*_0_*S*_0_ (abscissa) versus *N*_0_*S*_90_ conditions (ordinate). Those differences were negligible when the signal was at 0°, but were appreciably greater when the signal was at 90°, averaging 4.7 dB for the 42-dB masker and 9.8 dB for the 72-dB masker. An ANOVA of peak thresholds minus dip thresholds showed significant main effects of signal location (*F*_(1,7)_ = 55, *p* < 0.001) and masker level (*F*_(1,7)_ = 25, *p* = 0.0016), and significant two-factor interaction of signal location and masker level (*F*_(1,7)_ = 10.6, *p* = 0.014).

The *combined* masking release given by dip placement of the signal and 90° separation of signal and masker is given by the threshold in the peak *N*_0_*S*_0_ condition minus that in the dip *N*_0_*S*_90_ condition. That value averaged 27.5 dB (s.d. = 2.5 dB) for the 72-dB masker level and 22.5 dB (s.d. = 3.5 dB) for the 42-dB level. At both levels, the combined masking release was greater than that due to masker timing in the *N*_0_*S*_0_ condition *plus* the SRM in the peak condition: 18.6 dB (s.d. = 2.9 dB) at the higher masker level and 18.8 dB (s.d. = 3.6 dB) at the lower level. The differences between combined and summed releases were 8.9 dB at the higher masker level and 3.7 dB at the lower level, a significant difference between masker levels (*t*-test: *t*_7_ = 3.3, *p* = 0.014).

The present open-field results largely confirm expectations based on the closed-field results of Buss *et al*. (2003). In the open field, envelope and spatial effects on masking release were supra-additive, and that supra-additivity increased with increasing masker level. Analogous to the closed-field study, the differences between peak and dip thresholds were negligible in the *N*_0_*S*_0_ spatial condition at both low and high masker levels, increasing in the *N*_0_*S*_90_ condition. Also analogous to the close-field study, differences in thresholds between peak and dip conditions increased in the *N*_0_*S*_90_ spatial condition, although that was seen at a lower masker level than in the closed-field study. That is, SRMs averaged 4.7 dB at the lower masker level, compared to essentially zero difference in the closed-field study, and averaged 9.8 dB in the *N*_0_*S*_90_ condition, about 5 dB greater than the peak differences in the *N*_0_*S_π_* condition in the closed-field study. The magnitudes of SRM in the peak condition of the present study (17.8 and 17.7 dB at low and high masker levels, respectively) were slightly greater than the magnitudes of BMLD observed under headphones in the peak condition by Buss *et al*. (2003) (∼14 and 15 at the corresponding masker levels). In contrast, SRM in the dip condition of the present study (21.5 and 26.6 dB at low and high masker levels, respectively) was appreciably higher than the BMLD in the previous study (∼15 and 22.5 dB at the corresponding masker levels).

## V. GENERAL DISCUSSION

The goal of this study was to understand the degree to which spatial and envelope-related effects might combine in open-field conditions to improve detection of a tone in the presence of an on-signal masker. The work was inspired by previous studies conducted in closed-field (i.e., headphone) conditions (Buss *et al*., 2003; Epp and Verhey, 2009a,b). The present open-field results showed a greater magnitude of SRM than the BMLD seen in the previous closed-field studies and, in contrast, a lesser magnitude of envelope (i.e., CMR and masker timing) effects. At the higher of the two maskers level that were tested, SRM and CMR were additive, in agreement with the demonstration of superposition by Epp and Verhey (2009a), whereas SRM and CMR were sub-additive at the lower masker level. Release from masking due to temporal placement of a brief signal at a masker minimum (a dip) compared to that at a maximum (a peak) was strongly supra-additive with spatial release, even more so than predicted from the closed-field study by Buss *et al*. (2003). In all experiments, synergistic effects of envelope and spatial factors were greater at higher than at lower masker levels.

### A. Spatial masking release is greater than predicted from closed-field conditions

In typical closed-field studies of BMLD, an *N*_0_*S_π_* stimulus is produced by inverting the phase of the signal at the two ears, yielding an IPD of *π* radians in the signal. In the open field, given the dimensions of a typical human head, one would expect a 700-Hz tone presented from 90° azimuth also to produce an IPD of ∼*π* radians. For that reason, one might expect SRM to be about equal in magnitude to closed-field BMLD. The SRM that we measured, however, consistently was greater than that expected from a simple introduction of a *π*-radian IPD. Mean values of BMLD reported by Epp and Verhey (2009a) were only 12–13 dB, whereas our SRM values across both masker levels in Experiment 1A averaged about 4–9 dB greater, from 16.8 to 21.6 dB. The values of BMLD measured in the closed field in Experiment 1B (19.0 and 14.4 dB) were somewhat less than the corresponding SRM values, but still were greater than those in the Epp and Verhey (2009a) study. Mean values of BMLD reported by Buss *et al*. (2003) ranged from ∼14 to 22.5 dB, depending on masker level and masker timing condition, whereas our values of SRM in Experiment 2 were consistently 2–4 dB greater, averaging 17.7 to 26.6 dB at corresponding conditions.

We sought an explanation for the finding of open-field SRM that was greater than expected. In addition to the change in IPD of ∼*π*-radians, one would expect a 90° shift in the signal location to result in a change in the signal ILD from 0 to ∼4.2 dB and the signal-to-masker ratio at the near ear to increase by ∼2.6 dB; those values are from measurements from a KEMAR mannequin, available on-line (Gardner and Martin, 1995). Experiment 1B, in the closed field, evaluated the effects of such ILD and signal-to-masker changes on BMLD, using round numbers of 5 dB ILD achieved by a 2.5-dB increase in level at the right ear and 2.5-dB decrease at the left ear. In the *N*_0_*S*_0_ condition, a change in the signal ILD from 0 to 5 dB gave the expected result of consistently reduced detection thresholds; note that IPD = 0 radians and ILD = 5 dB is an artificial condition that would not normally be produced by an open-field source. In the *N*_0_*S_π_* condition, however, introduction of 5 dB ILD gave the unexpected result of no significant reduction of thresholds.

The influence of ILD on BMLD was studied in early work by Colburn and Durlach (1965) and by Egan (1965). The results of those studies most relevant to the present work are that addition of an ILD tended to depress thresholds (i.e., improve detection) in the *N*_0_*S*_0_ condition but tended to elevate thresholds (i.e., impair detection) in the *N*_0_*S_π_* condition. Those results were fit well by Durlach's Equalization and Cancellation model (Durlach, 1963). Put simply, in the *N*_0_*S*_0_ condition, an ILD would introduce a difference in the otherwise equal signal-plus-noise stimuli at the two ears, thereby facilitating signal detection. In the *N*_0_*S_π_* condition, the reduction in sound level at the attenuated ear would weaken the interaural difference produced by the antiphase signal. Regarding the present closed-field (Experiment 1B) results, the present observation of a significant depression of threshold by the addition of an ILD in the *N*_0_*S*_0_ condition accords with the previous reports (Colburn and Durlach, 1965; Egan, 1965), plus there would have been some additional depression of nominal threshold due to the 2.5-dB increase in signal-to-masker ratio. The lack of a significant effect of the added ILD on the *N*_0_*S_π_* thresholds can be explained by effects of the elevated threshold (or decreased BMLD) reported previously (Colburn and Durlach, 1965; Egan, 1965) working in opposition to the depression of threshold due to the increased signal-to-masker ratio. The overall reduction in the present BMLD in the 5- versus the 0-dB ILD conditions is explained simply by the depression of thresholds by the ILD in the *N*_0_*S*_0_ condition paired with the absence of significant threshold change in the *N*_0_*S_π_* condition.

Although our closed-field BMLD results from Experiment 1B fit well with previous closed-field BMLD studies, they do not provide a satisfactory explanation for the greater magnitude of open-field SRM that was observed compared to closed-field BMLD. That discrepancy clearly warrants further study.

### B. CMR is relatively weak in these open-field conditions

We are unaware of previous measures of classical CMR using open-field stimuli, particularly any that permitted direct comparisons between open-field and closed-field conditions. In the open-field measures of CMR in Experiment 1A, we observed prominent CMR in most masker-level and spatial conditions, although the magnitude of CMR consistently was less than that obtained in the closed field by Epp and Verhey (2009a,b). The magnitude of CMR in our own closed-field results (Experiment 1B) was intermediate between our open-field results and the closed-field results reported by Epp and Verhey (2009a,b). The masker levels in our experiments were different from the level used in the previous study, but our levels of 42 and 72 dB SPL bracketed the 60-dB-SPL level used by Epp and Verhey. Our open-field CMR magnitudes were 3.7–5.0 dB at the higher masker level and 1.9–7.5 dB at the lower level compared to 9–10 dB reported by Epp and Verhey (2009a,b). One might be tempted to attribute the reduced CMR in some way to the enhanced SRM that we observed. That explanation might explain the very low levels of CMR obtained in the *N*_0_*S*_90_ condition at the lower masker level, averaging only 1.9 dB. Enhanced SRM, however, would not explain the observation that our CMR levels were reduced even in the *N*_0_*S*_0_ condition. Inspection of Fig. 2 at the lower masker level gives the impression that the *N*_0_*S*_90_ thresholds are approaching a floor, perhaps related to the threshold for detection of the unmasked signal (e.g., Moore and Shailer, 1991). We note, however, that all the *N*_0_*S*_90_ thresholds are at least 20 dB above the unmasked-signal thresholds.

In contrast to CMR, the envelope release obtained by placing a brief stimulus at a minimum in the masker envelope was rather similar between the present open-field results (Experiment 2) and the closed-field study after which it was patterned (Buss *et al*., 2003). At the higher masker level, the previous closed-field study showed thresholds that were about equal for peak and dip in the *N*_0_*S*_0_ condition and 8 dB lower for dip than peak in the *N*_0_*S_π_* condition. Similarly, in the open field, we obtained only minor peak-to-dip differences (averaging 0.9 dB) for the frontally located signal (*N*_0_*S*_0_) whereas dip thresholds were 9.8 dB lower than peak when the signal source was shifted to 90° (*N*_0_*S*_90_). At the lower masker level, Buss *et al*. (2003) obtained nearly equal dip and peak thresholds in the *N*_0_*S_π_* condition in five of six listeners, but a difference of about 10 dB in the sixth listener. In our open-field measures at the lower masker level, the peak-to-dip differences were 4 dB or greater in six of eight listeners, averaging 4.7 dB across all eight listeners.

### C. Sub-additivity, superposition, and supra-additivity of masking release

Previous studies have shown a variety of results with regard to combined release from masking by envelope and spatial factors, including additivity/superposition, sub-additivity, and supra-additivity. One of the results that prompted the present study was the demonstration of superposition of CMR and BMLD by Epp and Verhey (Epp and Verhey, 2009a,b; Epp *et al*., 2013). In that work, on average, the masking release due to combined CMR and BMLD was equal to the sum of masking release by either factor alone, the magnitude of CMR was largely independent of signal IPD, and BMLD was largely independent of comodulation. Such superposition was observed in the present study in open-field conditions only at the higher masker level (Experiment 1A). A number of other studies have demonstrated sub-additivity of CMR and binaural or SRM, in which CMR can be substantially reduced in *N*_0_*S_π_* or *N*_0_*S*_90_ compared to *N*_0_*S*_0_ conditions (Cohen and Schubert, 1985, 1991; Hall *et al*., 1988, 2011; the present Experiment 1A at the lower masker level and Experiment 1B). Indeed, there are examples of individual listeners who show essentially no CMR in *N*_0_*S_π_* or open-field *N*_0_*S*_90_ conditions [Hall *et al*., 1988; the present Figs. 2(B), 2(D), and 2(H) at the lower masker level].

Magnitudes of CMR can vary depending on stimulus and data-analysis conditions. Epp and Verhey (2009b) and Hall *et al*. (2011) have pointed out that CMR is greater when computed relative to a reference condition of uncorrelated flanking bands (as in the present study) compared to that using reference condition of no flanking bands. Also Hall *et al*. (2011) have shown a difference in additivity of CMR and BMLD depending on whether or not the masking noise is continuous or is gated on only during listening intervals (as in the present study). That is, thresholds tended to be lower in continuous than in gated maskers specifically in the *N*_0_*S*_0_ CM condition, resulting in a reduction in BMLD in that (comodulated, continuous masker) condition. Even in experiments that used equivalent conditions (e.g., gated maskers and CMR based on a reference of uncorrelated flanking bands), there can be noticeable differences between laboratories and among listeners. For instance, Epp and Verhey (2013) showed, on average, superposition of CMR and BMLD, but two of their ten listeners [depicted in Epp and Verhey (2013), Figs. 3(e) and 3(f)] show prominent sub-additivity, and three of the nine listeners in the study by Hall *et al*. (2011) showed superposition or slight supra-additivity of CMR and BMLD despite an average finding of sub-additivity.

Supra-additivity of envelope and binaural/spatial effects have been demonstrated as enhanced masking release in *N*_0_*S_π_* or *N*_0_*S*_90_ conditions during brief periods of elevated signal-to-masker ratio (on a time scale of ∼10 ms; Buss *et al*., 2011). Grose and Hall (1998) showed that listeners give preferential weight to epochs of low masker energy in *N*_0_*S_π_* but not *N*_0_*S*_0_ conditions, implying a positive synergy of envelope and binaural effects. Hall *et al*. (1998) showed that BMLD consistently is greater in conditions of high-fluctuation compared to low-fluctuation narrowband noise. In that study, however, only three of six listeners showed enhanced BMLD due to decreased *N*_0_*S_π_* thresholds; in other cases, the enhanced BMLD resulted from elevated *N*_0_*S*_0_ thresholds.

Buss *et al*. (2003, 2007) showed that signal detection was enhanced when a brief signal was placed in time at a masker-envelope minimum (a dip) compared to placement at an envelope maximum (a peak), but that effect was observed only in *N*_0_*S_π_* conditions, not *N*_0_*S*_0_. Similarly, in Experiment 2, we observed substantial benefits of dip placement of brief signals in *N*_0_*S*_90_ but not *N*_0_*S*_0_ conditions. That is, SRM and a feature of the masker envelope were strongly supra-additive. Buss *et al*. (2003; their Fig. 6) showed that, at threshold signal-to-masker levels, addition of an antiphase (*S_π_*) signal to a diotic noise substantially degraded interaural correlation when the signal was added to a masker minimum, whereas the added antiphase signal had negligible effect on interaural correlation when the signal was added to a masker maximum. A substantial body of research supports the notion that detection of an antiphase signal in a diotic noise reflects detection of a disruption of interaural correlation by the antiphase signal (reviewed by Bernstein and Trahiotis, 1996). Signal detection based on interaural decorrelation could account for enhanced signal detection (and enhanced BMLD and SRM) in dip compared to peak conditions shown by Buss *et al*. (2003) and seen in the present study. In contrast, addition of an in-phase signal would have no effect on interaural correlation of diotic noise, regardless of dip or peak condition, which supports the observation of essentially no difference between dip and peak condition for the in-phase signal in the previous closed-field study (Buss *et al*., 2003) or for the frontal signal in the present study.

Buss and Hall (2011) suggested that the enhanced benefits of *N*_0_*S_π_* binaural conditions for detection of a brief signal in the presence of highly fluctuating maskers might also reflect a release from informational masking. That is, in addition to the energetic masking caused by the overlap of signal and masker spectra, there might be some informational masking due to confusion of the brief signal with fluctuations of the masker. The well-known spatial or binaural release from informational masking (Kidd *et al*., 1998) might mitigate that confusion. Yet another additional explanation is suggested by results of a study of IPD sensitivity at various phases of amplitude modulation (Dietz *et al*., 2013). That study showed that listeners' use of interaural fine structure for a lateralization judgment is limited to the rising segment of amplitude modulated sounds. One could posit that a signal placed at the minimum of a masker envelope, or within deeply fluctuating maskers in general, might offer better glimpses of the rising phases of brief signal envelopes and, therefore, could enhance the use of temporal fine structure in formation of distinct masker and signal-plus-masker objects.

Previous studies have shown that BMLD for detection of a tone in a narrow-band masker increases with increasing masker level (Hall *et al*., 1983; Hall and Harvey, 1984; Nitschmann *et al*., 2009), and CMR has been shown to increase with increasing masker level (Moore and Shailer, 1991). Also, Buss *et al*. (2003, 2007) found supra-additivity of masker timing and BMLD primarily at the highest masker level. In the present study, greater positive synergy between envelope and spatial release was observed at higher than at lower masker levels. That is, in Experiment 1A, the combination of CMR and SRM shifted from sub-additivity to additivity with increasing masker. In Experiment 2, masker release due to placement of the signal at a masker dip and SRM were somewhat supra-additive at the lower masker level and became significantly more supra-additive at the higher level. Stated another way, SRM tended to increase with increasing masker level primarily in CM or dip conditions, and CMR or release in the dip condition tended to increase with increasing level only in the *N*_0_*S*_90_ condition.

## VI. CONCLUDING REMARKS

The present results demonstrate that both masker envelope fluctuation and spatial separation of signal and masker could enhance signal detection in a open field. In nearly every instance, the combined masking release due to envelope-related and spatial effects was greater than the greater of the two effects alone. Combined comodulation and spatial masking release typically was equal or less than the sum of CMR and SRM (or BMLD). Placing a brief signal at a dip rather than at a peak of the masker envelope, however, generally enhanced SRM, resulting in substantial supra-additivity of envelope-related and spatial masking release.

The present study also demonstrates that extrapolation from the closed field to the open field is not as straightforward as one might expect. We consistently observed greater masking release in the spatial *N*_0_*S*_90_ condition than is reported in the closed-field *N*_0_*S_π_* condition, and the masking release that we observed due to envelope-related effects generally was less than that observed under headphones. Closed-field conditions provide the opportunity to manipulate monaural and binaural stimulus features independently and analytically, whereas results obtained in an open sound field arguably provide a closer approach to real-world listening. The present study suggests a need for further direct comparisons of closed-field and free-field hearing.
